# Supplementary figures and images for: TbsNet: the importance of thin-branch structures in CNNs
Source: PeerJ Comput Sci. 2023 Jun 16;9:e1429. doi: 10.7717/peerj-cs.1429 (PMC10280644; doi:10.7717/peerj-cs.1429)

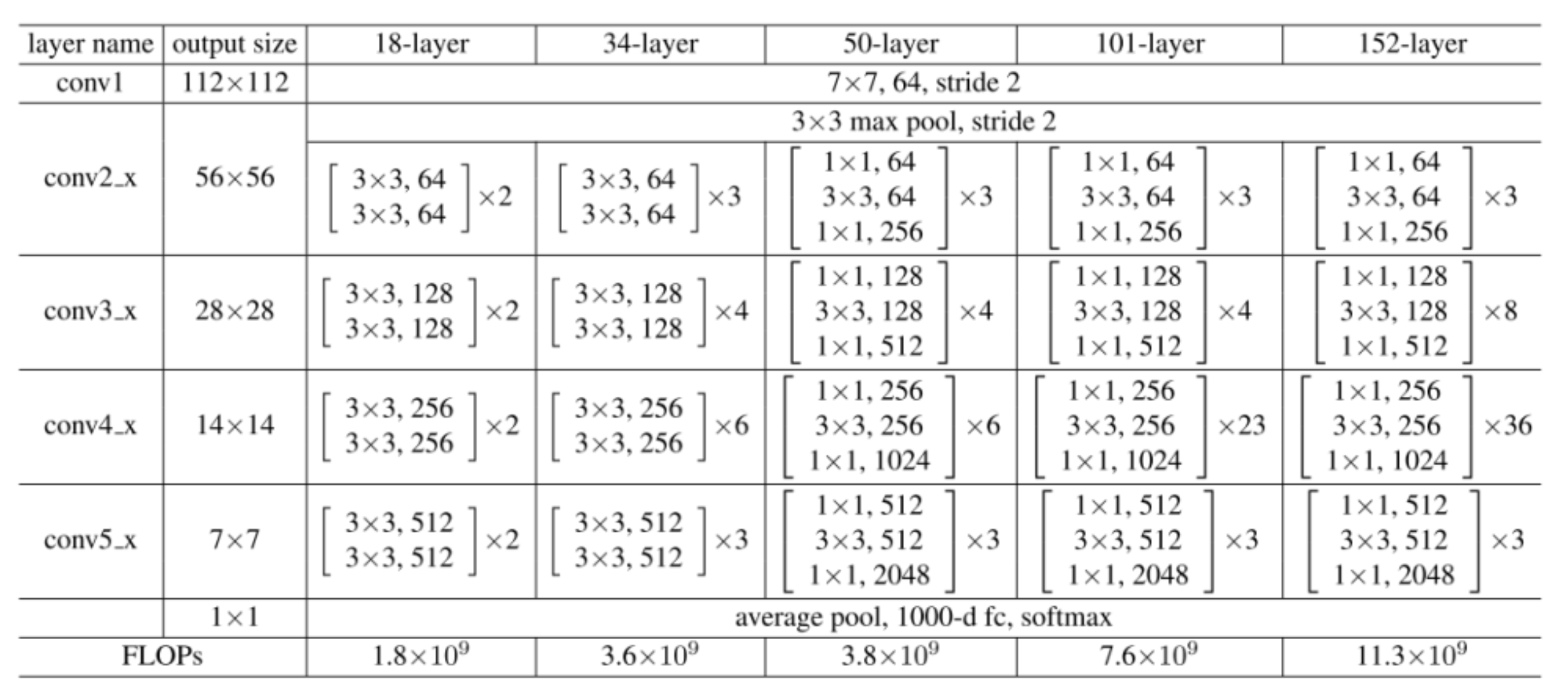

Supplement: Supplemental Information 1 — The training and testing programs on the CIFAR-10, CIFAR-100, Tiny-ImageNet, ImageNet-1K data sets [file peerj-cs-09-1429-s001.zip › source_code/TbsNet-Cifar/tools/resnet╜ß╣╣═╝.png]

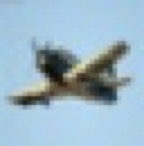

Supplement: Supplemental Information 1 — The training and testing programs on the CIFAR-10, CIFAR-100, Tiny-ImageNet, ImageNet-1K data sets [file peerj-cs-09-1429-s001.zip › source_code/TbsNet-ImageNet/picture/test_img_2.png]

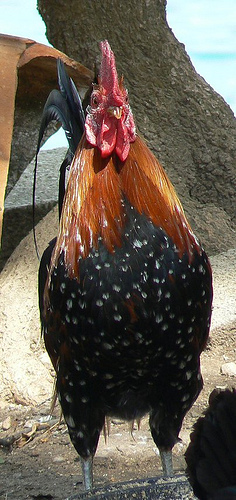

Supplement: Supplemental Information 1 — The training and testing programs on the CIFAR-10, CIFAR-100, Tiny-ImageNet, ImageNet-1K data sets [file peerj-cs-09-1429-s001.zip › source_code/TbsNet-ImageNet/test/n01514668_115.JPEG]
